# Could SARS-CoV-2 herald a surge of multiple sclerosis?

**DOI:** 10.1186/s41983-021-00277-5

**Published:** 2021-02-10

**Authors:** Nevin Mohieldin Shalaby, Hatem Samir Shehata

**Affiliations:** Neurology Department, Kasr Al-Ainy School of Medicine, Cairo University, Department No.15, Second Floor, Al-Saraya St., Al-Manial District, Cairo, Egypt

**Keywords:** COVID-19, SARS-CoV-2, Multiple sclerosis, Neurotropism

## Abstract

During the current COVID-19 pandemic, many queries are raised regarding its nature, outcome, and sequelae. This letter raises the concern of potential impact on increasing the incidence of multiple sclerosis whose pathology involves a possible viral etiology. Besides, the potential neurotropism of the acute respiratory distress syndrome corona virus-2 (SARS-CoV-2), which is still not established, may raise concerns about the use of certain disease modifying therapies namely natalizumab.

To the editor

## Introduction

The evidence that viruses are associated with multiple sclerosis (MS) stems from various experimental studies [1].In the context of the current coronavirus disease-19 (COVID-19) pandemic, it is worth searching and digging deep in the history of human coronaviruses (HCoVs) to find whether they might be related to MS pathology. HCoVs are responsible for 10–35% of common cold in humans [2]. COVID-19 is caused by severe acute respiratory syndrome coronavirus-2 (SARS-CoV-2), a beta-coronavirus showing similarities with SARS-CoV. SARS-CoV-2 binds to angiotensin-converting enzyme-2 receptor whose expression is ubiquitous, including the central nervous system (CNS) [3].

## Main text

There is a proven association between HCoV and MS pathogenesis which has evolved from several experimental studies which revealed that murine coronavirus infection of susceptible mice led to an inflammatory demyelination similar to MS; coronavirus RNA sequences and its antigen were detected in demyelinating lesions [4]; two HCoV strains were studied in the brains of patients with MS (pwMS); HCoV-229E viral RNA was detectable in the CNS of 36% pwMS and in none of those with other neurological diseases and normal controls, whereas no HCoV-OC43 nucleic acid was detected in any of the specimens; and HCoV-myelin cross-reactive T cell lines were predominantly found in pwMS compared to patients with other neurological diseases or healthy controls. Moreover, the cross-reactivity observed appears to be found in MS but not in other inflammatory or neurological disorders. Molecular mimicry could be the mechanism through activation of myelin-reactive T cells by a virus infection in a genetically predisposed individual [1].

Furthermore, coronavirus neurotropism has been demonstrated in humans; an acute infection by HCoV was detected in cultures of human microglia, astrocytes, and oligodendrocytes; also, a persistent infection was demonstrated in cell lines from nervous system [1]. Neurological manifestations have been reported as a result of SARS-CoV-2 systemic infection, and the access can be either hematological or neuronal via olfactory nerve [5]; however, neurotropism is not established yet for the SARS-CoV-2.

Based on the aforementioned data, during the current COVID-19 pandemic and the emergence of the corona virus strains, two concerns are worth considering; the possibility of increasing MS, as well as other neurological autoimmune disorders, incidence worldwide in the coming years; however, no solid conclusions can be drawn regarding this possibility, as different strains can exhibit various characteristics as evidenced by the probable association of HCoV-229E to MS pathology and the absence of such association in the HCoV-OC43 strain [1], and still the behavior of SARS-CoV-2 is being explored; the other concern is reconsideration of the use of natalizumab, which is considered relatively safe being not a cause of peripheral lymphopenia, but inducing its effect centrally by preventing activated auto-reactive T cell trafficking into the CNS [6], meanwhile, we may be faced by a neurotropic virus whose effects may be revealed in the coming future.

## Conclusion

The full characterization of SARS-CoV-2 and its influence on the nervous system and potential triggering of autoimmune CNS inflammatory diseases, including MS, represent a rich field for research at the present time and in the future to follow its potential sequelae.

## Data Availability

Not applicable

## Abbreviations

MS: Multiple sclerosis
COVID-19: Coronavirus disease-19
HCoVs: Human coronaviruses
SARS-CoV-2: Severe acute respiratory syndrome coronavirus-2
RNA: Ribonucleic acid
pwMS: Patients with MS
